# Hemostatic Responses to Multiple Bouts of Firefighting Activity: Female vs. Male Differences in a High Demand, High Performance Occupation

**DOI:** 10.3390/ijerph19042124

**Published:** 2022-02-14

**Authors:** Denise L. Smith, Gavin P. Horn, Steven J. Petruzzello, Gregory G. Freund, Samuel I. Bloom, Bo Fernhall

**Affiliations:** 1 Illinois Fire Service Institute, University of Illinois Urbana-Champaign, Champaign, IL 61820, USA; gavin.horn@ul.org (G.P.H.); petruzze@illinois.edu (S.J.P.); freun@illinois.edu (G.G.F.); fernhall@uic.edu (B.F.); 2 Department of Health and Human Physiological Sciences, Skidmore College, Saratoga Springs, NY 12866, USA; sambloom28@gmail.com; 3 Fire Safety Research Institute, Underwriters Laboratories Inc., Columbia, MD 21045, USA; 4 Department of Kinesiology and Community Health, University of Illinois at Urbana-Champaign, Urbana, IL 61820, USA; 5 Division of Nutritional Sciences, University of Illinois at Urbana-Champaign, Urbana, IL 61820, USA; 6 Department of Nutrition and Integrative Physiology, University of Utah, Salt Lake City, UT 84112, USA; 7 Department of Kinesiology and Nutrition, University of Illinois at Chicago, Chicago, IL 60612, USA

**Keywords:** hemostasis, coagulation, fibrinolysis, gender

## Abstract

While the fire service has long been a male-dominated occupation, women’s participation in this strenuous, high risk, high performance activity has increased in recent years. Firefighting induces significant cardiovascular strain, including hemostatic disruption; however, the effect of sex on hemostatic responses has not been investigated despite evidence that there are sex-related differences in hemostatic variables at rest and following exercise. Thus, we investigated hemostatic responses in age- and BMI-matched male and female firefighters who performed 3–4 evolutions of firefighting drills over a 3 h period. Venous blood samples were collected before and after the firefighting training drills and hemostatic variables were assessed. Firefighting significantly increased platelet count and factor VIII, tissue plasminogen activator (t-PA) antigen, and t-PA activity, and decreased activated partial thromboplastin time and plasminogen activator inhibitor (PAI-1) activity. Females had lower values for epinephrine-induced platelet closure time, antithrombin III, PAI-1 activity, and PAI-1 antigen. There were no interactions between sex and time for any variables assessed. In conclusion, multiple bouts of firefighting activity resulted in a procoagulatory state. Although there were sex differences for several hemostatic variables, male and female firefighters did not differ in their hemostatic response to multiple bouts of firefighting.

## 1. Introduction

Firefighters represent a unique occupational group that routinely perform dangerous work to protect their communities. While firefighters face multiple occupational risks, it is noteworthy that approximately 50% of duty-related deaths among firefighters are due to sudden cardiac events (SCE) [1]. The fire service has long been a male-dominated occupation, but women’s participation has increased in recent years. Based on data submitted to the US Fire Department profile, it has been reported that there are 12,850 women career firefighters and 72,250 women volunteer firefighters in the US, comprising 7.3% of the US fire service overall [2]. Given our understanding of the physiological strain of firefighting and the (finally) increasing number of female firefighters, there is a pressing need to understand the hemostatic responses to firefighting. A better understanding of hemostatic changes may provide a mechanistic link to the increased risk of SCE following fire suppression work and how these responses may differ between male and female firefighters.

Although some studies on hemostatic responses to firefighting have included a small number of female firefighters [3,4], they were underpowered to determine the effect of sex on coagulation and fibrinolysis responses to firefighting. Thus, the effect of sex on hemostatic responses to firefighting remains unknown. Differences in platelet activation, coagulation, and fibrinolysis have been found between males and females at rest [5] and in response to an acute bout of exercise [6,7,8,9,10]. However, in part due to the historically low number of female firefighters in the profession, women are often excluded from studies investigating the physiological effects of firefighting and to date, no studies have provided a comparison between male and female hemostatic responses to firefighting activities.

Previous studies found that acute bouts of short duration firefighting increases platelet number, aggregability [3,11], and coagulatory potential [4,12,13]. These factors might partially explain the heightened risk of SCE during or shortly after firefighting activity. Prolonged operations consisting of multiple bouts of firefighting activity (often defined by using one air bottle, or approximately 12–20 min) can lead to greater physiological strain (e.g., higher heart rate and core temperature) than short-term firefighting [14]. However, to our knowledge there are no published data regarding the effect of multiple bouts of firefighting activities on hemostatic balance. The purpose of this study was to determine the effects of multiple bouts of firefighting on hemostatic responses and to compare those responses in male and female firefighters. We hypothesized that multiple bouts of firefighting would lead to a procoagulatory response in both male and female firefighters, and that females would experience a greater procoagulatory shift than men.

## 2. Materials and Methods

Participants were 34 firefighters (19 male, 15 female) from career and volunteer fire departments across the state of Illinois. Participants were current firefighters between the ages of 23 and 48 years, in active service, and were medically cleared to serve as a firefighter by their home department. This is a secondary analysis from a larger study that investigated cardiovascular and thermal responses to firefighting [14]. For this analysis, we included all female firefighters and then matched a subset of male firefighters based on age and body mass index. Prior to taking part in the study, participants were fully informed of the purposes of the study and provided written informed consent. This study was approved by the University of Illinois Institutional Review Board.

Participants were provided with a standardized meal (energy bar, protein drink, and banana), which was consumed approximately 1 h prior to pre-firefighting data collection. During firefighting activities, participants were encouraged to consume fluids ad libitum to maintain hydration. During breaks in training, doffing of gear was also encouraged to promote cooling, and rest and rehydration was provided.

Firefighters performed 3–4 evolutions of live-fire training exercises in a concrete and steel training building, with fires fueled by pallets and straw burners, over an approximate 3 h period. Firefighters were instructed to complete the tasks at a typical fireground pace.

Firefighting drills varied throughout the 3 h training period, but included up to four specific evolutions typically lasting 15–30 min separated by 20–40 min of rest (recovery). The training evolutions included coordinated fireground operations to suppress fires and search for victims in four scenarios: a basement fire, a restaurant, a single-family dwelling, and a multistory dwelling. During recovery, firefighters hydrated, cooled, reviewed/critiqued the evolution with instructors, and refilled their air cylinders. Firefighters wore their own department-issued full firefighting personal protective equipment and self-contained breathing apparatus during all firefighting activities. Data were collected during the summer and fall months when the ambient temperature ranged between 15 °C and 25 °C.

Blood samples were obtained immediately before and approximately 10–30 min after the completion of firefighting activities. Blood samples were obtained using tubes containing 3.2% sodium citrate for measurements of all coagulation and fibrinolytic factors, except for the assessment of tissue plasminogen activator (tPA) activity, for which samples were collected into Stabilyte tubes (Biopool, Wicklow, Ireland). For analyses other than platelet number and function, samples were centrifuged at 2300× *g* rpm for 25 min at 4 °C with the plasma removed and placed into 12 aliquots and stored at ~70 °C for subsequent analysis. Both tPA activity and plasminogen activator inhibitor (PAI-1) activity were analyzed in duplicate using commercially available chromogenic substrate kits (Diapharma Group, Inc., West Chester, OH, USA). PAI-1 antigen, tPA antigen, and fibrinogen were analyzed in duplicate using an enzyme-linked immunosorbent assay kit (ELISA; American Diagnostica, Stamford, CT, USA). Activated partial thromboplastin time (aPTT) was analyzed using STA-PTT Automate 5 (Diagnostica Stago, Inc., Parsippany, NJ, USA). Antithrombin III was analyzed in duplicate using an ACTICHROME chromogenic activity kit (American Diagnostica, Stamford, CT, USA). Factor VIII activity was analyzed in duplicate using a chromogenic assay kit (Chromogenix/Diapharma Group Inc., West Chester, OH, USA). Platelet count was assessed from venous whole blood as part of a complete blood count analysis (COULTER LH 700 Series; Beckman Coulter, Inc., Fullerton, CA, USA). Platelet function was assessed by epinephrine-induced and adenosine 5′-diphosphate-induced platelet aggregability using a platelet function analyzer (PFA-100; Dade Behring, Deerfield, IL, USA). Venous blood samples were maintained at room temperature and analyzed within 2 h of collection. Plasma volume changes as a result of firefighting were determined according to the methods of Dill and Costill [15].

Heart rate was measured throughout the firefighting drills using a heart rate watch (S625X, Polar Electro Oy, Oulu, Finland). Core temperature was measured using a gastrointestinal temperature transmitter pill that was ingested 6–12 h prior to the drills (Mini-Mitter Vital Sense; Phillips Respironics, Bend, OR, USA). Unfortunately, some data loss occurred in both heart rate and core temperature due to strenuous activity dislodging the heart rate strap as well as some “lost” core temperatures pills near the beginning of the scenarios, loss of communications with sensors during data collection, and water ingestion impacting some core temperature capsules. For these comparisons, we only report data from participants who had valid heart rate and core temperature data throughout the test scenario. Nude body weight was determined pre- and post-firefighting activities with a digital scale and recorded to the nearest 0.1 kg. Standing height was measured with a stadiometer and recorded to the nearest 0.5 cm.

Results are expressed as mean (standard deviation). Variables were checked for normal distribution using Shapiro–Wilk tests, and those variables not normally distributed were log transformed (natural logarithm) prior to statistical analyses. Differences in descriptive variables between sexes were evaluated with an independent *t*-test. To evaluate the effect of firefighting activity and sex differences in response to firefighting, a 2 × 2 (sex [male vs. female] by time [pre vs. post firefighting]) analysis of variance with multiple measures was conducted. Analyses were performed in SPSS (v. 23 IBM, Armonk, NY, USA) with significance set at an alpha of 0.05.

## 3. Results

Male and female firefighters were matched for age and body mass index; however, male firefighters were taller (*p* = 0.001) and heavier (*p* = 0.002) than female firefighters (Table 1). There was no effect of sex on heart rate, and heart rate increased significantly during firefighting (*p* < 0.001), reaching a peak of approximately 183 bpm in males and 187 bpm in females (Table 2). Core temperature increased significantly, reaching a peak value (the highest recorded value during or after completion of firefighting activities) of 38.8 °C in females and 39.0 °C in males. There was no main effect of sex for core temperature and no significant sex by time interaction, but a greater elevation in core temperature for males than females approached significance (*p* = 0.055). There was no significant difference in plasma volume or body weight change between sexes. Body weight for female firefighters decreased 0.67 kg from pre- to post-testing, while the male firefighters lost 1.11 kg.

Table 3 presents platelet count, coagulatory responses, and fibrinolytic responses to firefighting activities for male and female firefighters. Platelet count increased significantly following firefighting activity (*p* < 0.001); however, there was no main effect of sex (*p* > 0.05). There was no time main effect detected for platelet closure time, but a significant sex effect for the epinephrine treated samples was detected (*p* = 0.016). There was a significant effect of time for aPTT (*p* < 0.001), with aPTT being shorter following firefighting activity. A significant main effect of time for factor VIII (*p* = 0.002) was observed. There was no effect of time on antithrombin III (*p* > 0.05); however, male firefighters had significantly higher levels of antithrombin III than their female counterparts (*p* = 0.001). There was a main effect for time (*p* < 0.001) for t-PA antigen, with t-PA antigen increasing following firefighting, and a nearly significant effect for sex (*p* = 0.067) (with women having lower values at rest and after firefighting). There was a main effect of time for t-PA activity (*p* < 0.001). Firefighting had a significant time main effect on PAI-1 antigen (*p* < 0.05), with male firefighters having higher (but not statistically significant) PAI-1 antigen levels than female firefighters. PAI-1 activity was higher in male firefighters than female firefighters (*p* = 0.018) and lower after firefighting activity (*p* < 0.001).

## 4. Discussion

The present study extends previous research on hemostatic responses to performing strenuous activity/work by examining the effects of multiple bouts of firefighting activities on markers of coagulation and fibrinolysis in both males and females. The main findings are that (a) multiple bouts of firefighting caused a shift towards a procoagulatory state and (b) sex did not affect the hemostatic responses to firefighting, as evidenced by a lack of significant time by sex interactions.

Women are increasingly joining the fire service with current estimates that approximately 8% of the US fire service is made up of women. Firefighting involves strenuous muscular work requiring high levels of strength, aerobic fitness, and anaerobic power, and resulting in significant cardiovascular strain that may trigger sudden cardiac events in vulnerable individuals [16]. The present study provided the first detailed investigation of the effect of sex on coagulation and fibrinolysis following firefighting, as little research exists on the hemostatic responses in female firefighters [3,4]. This study found differences between the sexes for several variables; however, hemostatic responses to firefighting activity did not differ between male and female firefighters. Direct comparisons of fibrinolytic responses to exercise in healthy males and females are scarce. Two early studies demonstrated that females had a greater fibrinolytic response to moderate exercise than males, as assessed by euglobulin lysis time [7,8]. In contrast, in a study that examined the effect of different exercise intensities in 20 (10 males, 10 females) healthy, untrained subjects aged 22 to 34 years and not taking medications, the fibrinolytic response was lower in females than males, particularly following maximal exercise [10]. Additionally, aPTT was significantly shorter for males compared with females at the end of both aerobic and anaerobic exercise.

Increased coagulatory potential represents a possible mechanism to explain the increased risk of sudden cardiac events during or following firefighting activities [16,17]. Several studies have examined the acute effects of firefighting on hemostasis and found that firefighting increases platelet aggregability and enhances coagulation as well as fibrinolysis, but ultimately leads to a procoagulatory state [12,13,18]. Yet, at actual firefighting events and during regular training, firefighters often perform intermittent bouts of activity over a prolonged time frame. These activities are characterized by strenuous physical work, followed by periods of rest/recovery to rehydrate, refill air cylinders, and to debrief. Research has found that multiple, intermittent bouts of firefighting are more physiologically demanding than a single acute bout, as core temperature increases to a greater extent [14]. In the current study, firefighters experienced a greater increase in core temperature on average than typically reported following firefighting activity [12,13,18]. Both an increase in core temperature and strenuous exercise are known to increase coagulation [19,20,21,22,23,24].

In the present study, platelet count increased approximately 21% following multiple bouts of firefighting, independent of sex. Previous research on the effects of a single acute bout of firefighting has reported an increase in platelet count of 17% to 29% [12,18]. The magnitude of the increase in platelet count is greater than what can be explained by hemoconcentration given the modest plasma volume changes we documented in this study. Importantly, however, during the prolonged training period firefighters were repeatedly encouraged to consume water. Dehydration may be more likely to occur in emergency operations where rehydration is not prioritized and this could lead to a further increase in platelet count and blood viscosity. The current study found a significant decrease in aPTT indicating a procoagulatory state for both male and female firefighters. The aPPT was shortened by 9.0% and 7.7% for female and male firefighters, respectively, which is similar in magnitude to changes reported for normal weight (12.2%) and obese (8.2%) male firefighters following a single bout of firefighting activity [18]. Similarly, we reported a 9.6% decrease in aPTT in a group of older male firefighters following a single bout of firefighting activity [12]. Collectively, these data suggest that strenuous firefighting results in a procoagulatory change as evidenced by a shortened aPTT, which is a global marker of coagulatory potential, and that the magnitude of the change is approximately 8–12% across a wide range of participant characteristics and training types.

Strenuous exercise is known to increase coagulatory potential and fibrinolysis [19,20,22,23,24,25,26,27]. In the present study, multiple bouts of firefighting resulted in increased coagulation and increased fibrinolysis. These data are congruent with previous research on a single bout of firefighting, which has shown similar increases in fibrinolytic activity in normal or overweight firefighters and in older firefighters, immediately following a single bout of firefighting [12,18]. Collectively, these findings suggest that firefighting activities, whether a single bout or multiple bouts, simultaneously increase coagulatory and fibrinolytic potential; however, the overall effect is procoagulatory based on the global measure of aPTT. Importantly, however, studies to date have included firefighters who were apparently healthy and had been cleared for duty by their home department. It remains unclear if firefighters with cardiovascular disease risk factors or underlying cardiovascular disease would respond to firefighting activities in a similar way.

## 5. Conclusions

In conclusion, multiple bouts of firefighting activities lead to increased coagulation and fibrinolytic markers, and an overall procoagulatory state in both men and women. While sex differences in coagulation and fibrinolysis exist, hemostatic responses to firefighting were not different between male and female firefighters. Furthermore, these findings extend previous work on short-duration firefighting. However, the results do not suggest that multiple bouts of firefighting are associated with a greater coagulatory change than short-duration firefighting of similar intensity.

## Figures and Tables

**Table 1:** Descriptive statistics for female and male firefighters. Values presented as mean (standard deviation).

Variable	Female (*n* = 15)	Male (*n* = 19)
Age (yrs)	35.1 (7.5)	33.2 (7.4)
Body Mass (kg)	69.8 (8.5)	81.1 (10.4)
Height (m)	1.69 (0.07)	1.80 (0.01)
Body Mass Index (kg·m^2^)	24.6 (3.8)	25.0 (3.2)

**Table 2:** Heart rate, core temperature, and hydration changes in responses to firefighting activities for male and female firefighters. Values presented as mean (standard deviation).

**Variable**	**Female**		**Male**		**Main Effects**		**Interaction**
	**Baseline**	**Peak**	**Baseline**	**Peak**	**Time**	**Sex**	**Time × Sex**
Heart Rate (bpm) ^†^	74 (11)	187 (12)	66 (8)	183 (14)	<0.001	ns	ns
Core Temperature (°C) ^§^	37.0 (0.2)	38.8 (0.4)	36.8 (0.5)	39.0 (0.7)	<0.001	ns	ns (*p* = 0.055)
	**Pre/Post Change**		**Pre/Post Change**			**Sex**	
Plasma Volume ^#^	0.74 (4.90)		−1.77 (5.64)		–	ns	–
Body Weight (kg) ^#^	−0.67 (0.64)		−1.11 (0.87)		–	ns	–

^†^ 12 female/14 male ^§^ 12 female/16 male ^#^ 15 female/19 male.

**Table 3:** Platelet, coagulatory responses, and fibrinolytic responses to firefighting activities for male and female firefighters. Values presented as mean (standard deviation).

Variable	Female (*n* = 15) ^#^		Male (*n* = 19) ^#^		Main Effects		Interaction
	Pre	Post	Pre	Post	Time	Sex	Time × Sex
Platelet count ^†^	254.9 (66.2)	307.6 (73.8)	255.7 (58.5)	310.0 (63.1)	<0.001	ns	ns
ADP closure time (s)	86.1 (22.8)	92.6 (23.3)	89.3 (12.0)	93.3 (42.3)	ns	ns	ns
EPI closure time (s)	89.9 (36.3)	94.1 (22.1)	112.5 (28.4)	110.8 (36.9)	ns	0.016	ns
aPTT (s)	33.3 (3.6)	30.2 (3.8)	33.7 (3.5)	31.1 (3.2)	<0.001	ns	ns
Fibrinogen (mg/dL)	223.7 (53.9)	229.6 (61.2)	220.8 (49.7)	231.2 (43.2)	ns	ns	ns
Factor VIII (iu/mL)	57.9 (22.9)	127.8 (87.4)	61.8 (29.4)	118.5 (43.6)	0.002	ns	ns
Antithrombin III (%)	93.9 (17.9)	96.7 (20.7)	110.2 (12.3)	117.7 (14.4)	ns	0.001	ns
t-PA antigen (ng/mL)	4.4 (4.2)	7.9 (5.2)	5.0 (2.7)	10.2 (4.2)	<0.001	ns (0.067)	ns
t-PA activity (iu/mL) ^§^	0.7 (0.3)	1.9 (1.2)	0.6 (0.3)	2.2 (1.1)	<0.001	ns	ns
PAI-1 antigen (ng/mL)	14.5 (7.1)	20.1 (10.0)	21.5 (8.8)	21.1 (6.9)	0.041	ns (0.067)	ns (0.076)
PAI-1 activity (ng/mL)	6.4 (8.0)	2.6 (1.8)	12.1 (10.2)	4.5 (3.9)	<0.001	0.018	ns

ADP, adenosine5-diphosphate; EPI = epinephrine; t-PA = tissue plasminogen activator; PAI-1 = plasminogen activator inhibitor. ^#^ Unless otherwise indicated ^†^ 14 female/19 male ^§^ 14 female/17 male.

## Data Availability

The data presented in this study are available on request from the corresponding author. The data are not publicly available due to participants’ privacy concerns.
